# Age-Related Impairment of Quality of Joint Motion in Vibroarthrographic Signal Analysis

**DOI:** 10.1155/2015/591707

**Published:** 2015-02-23

**Authors:** Dawid Bączkowicz, Edyta Majorczyk, Krzysztof Kręcisz

**Affiliations:** ^1^Institute of Physiotherapy, Faculty of Physical Education and Physiotherapy, Opole University of Technology, 76 Prószkowska Street, 45-758 Opole, Poland; ^2^Laboratory of Immunogenetics and Tissue Immunology, Ludwik Hirszfeld Institute of Immunology and Experimental Therapy, Polish Academy of Sciences, 12 Rudolfa Weigla Street, 53-114 Wrocław, Poland; ^3^Institute of Physical Education, Faculty of Physical Education and Physiotherapy, Opole University of Technology, 76 Prószkowska Street, 45-758 Opole, Poland

## Abstract

Aging is associated with degenerative changes in articular surfaces leading to quantitative and qualitative impairment of joint motion. Therefore, the aim of this study is to evaluate an age-related quality of the patellofemoral joint (PFJ) motion in the vibroarthrographic (VAG) signal analysis. Two hundred and twenty individuals were enrolled in this study and divided into five groups according to age. The VAG signals were collected during flexion/extension knee motion using an acceleration sensor and described using four parameters (VMS, P1, P2, and *H*). We observed that values of parameters VMS, P1, and P2 increase in accordance with the age, but *H* level decreases. The most significant differences were achieved between the youngest and the oldest participants' groups. Moreover, we show that parameters VMS, P1, and P2 positively correlate with age, contrary to negatively associated *H* parameter. Our results suggest that the impairment of joint motion is a result of age-related osteoarticular degenerative changes.

## 1. Introduction

A rising of life expectancy and declining births rate belong to the causes of aging of populations observed as unwelcome demographic changes in highly developed countries [1]. A senescence of the body occurs, among others, as the degenerative changes within locomotor system, which limit functionality of patients and lead to quality of life impairment [2]. Aging, particularly, relates to osteoarticular and ligamentous apparatus in a consequence of only limited ability to regeneration.

Among the typical age-related physiological processes in the joints are loss of ligaments elasticity, capsular fibrosis, and decline of muscles strength [3]. In addition, degenerated joints are often swollen due to increased production of synovial fluid with impaired quality. Senescence-associated mechanical changes are also observed, especially as the fibrillations process in the structures of articular surfaces covered by hyaline cartilage. Additionally, it is postulated that cartilaginous degeneration is initialized by age-related molecular alteration in both chondrocytes biology and cartilage matrix structure [4]. The latter often accompanies the articular cartilage degeneration, which may also contribute to the background of osteoarthritis pathogenesis. Among others, injuries and microinjuries, long-term immobilization, congenital and acquired disorders, and systemic inflammatory diseases can dramatically accelerate cartilage damage progression. The discussed mechanisms contribute to the cartilaginous disorders, which are associating with limited ability to the reduction of friction. As a result the impairment of joint motion quality with presence of grinding and cracking during articular motion is observed [5, 6]. Due to the specific biomechanical environment and significant weight-bearing, a patellofemoral joint (PFJ) as an integral element of the knee joint complex is remarkably exposed to this phenomenon [7].

A prevention as well as therapy of the disorders of osteoarticular apparatus based on an appropriate diagnosis is often limited to the medical interview, physical examination, and imaging by poor specific and low sensitive X-ray [8, 9]. For obtaining of the sufficient information of both early degenerative changes in bone and cartilage tissues and the soft tissues' condition more advanced imaging method, MRI, is proposed, but due to high expense it is available for limited patients group. Unfortunately, in all imaging methods only structural changes are observed and articular cartilage function and quality of joint motion are undetectable.

One of the methods described for assessment of function and articular cartilage motion quality is vibroarthrography (VAG). The mentioned examination by the registration of the vibroacoustic signals generated by motion of the articular surfaces could evaluate their function and joint motion quality directly [10]. It was shown that those VAG signals from abnormal joints possess different waveform pattern in comparison to those from healthy joints [11, 12]. This phenomenon seems to be a result of vibration magnitude increase in relation to articular cartilage degeneration. In physical examination during relating articular surfaces movement, it is observed as the crepitations (pathological mechanical vibration and acoustic signals) [13]. Due to the fact that crepitations are often observed for elderly individuals, the aim of this study is evaluation of quality of the PFJ motion and articular cartilage condition in further decades of life. According to the age-relative cartilage changes, vibroarthrographic signals interpretation may help to expand our knowledge about quality of motion during physiological senescence of the locomotor system.

## 2. Material and Methods

### 2.1. Study Population

A study material is consisted of randomly selected subjects who were participants in prevention program of the locomotor system dysfunctions realized at the Institute of Physiotherapy, Opole University of Technology, in 2012-2013 years. Accordingly to age patients were qualified to five groups (3th decade, 20–29; 4th decade, 30–39; 5th decade, 40–49; 6th decade, 50–59; 7th decade, 60–69 years old), each initially containing 70 persons. To prevent against any signal “artifact” of the PFJ disorders not associated with aging, based on the medical interviews and physical examinations, individuals with any diagnosed disorders of the knee, posttraumatic syndromes, neurological disorders, functional limitations (including any ability to flexion/extension motion in task with specified velocity), or feeling the pain were excluded from the study. Finally, to vibroarthrographic assessment 220 healthy individuals were enrolled in the study (127 women, 93 men), whose characteristics are shown in Table 1.

The project was approved by the Ethics Committee of Opole Voivodeship. Signed informed consent was obtained from all tested persons.

### 2.2. Vibroarthrographic Signal Assessment

For each knee, assessment of the PFJ function was performed with an acceleration sensor placed 1 cm above the apex of the patella using double-sided adherent tape. The vibroarthrographic evaluation of the PFJ quality of motion was based on a 6-second test in the sitting position, which included four times of (i) loose hanging legs with knees flexed at 90°; (ii) full knee extension from 90° to 0°; (iii) reflexion (from 0° to 90°). The constant velocities of both flexion/extension motion and measuring condition were kept at 82 beats per minute with a metronome. The angle of the knee joint was measured using an electrogoniometer which is placed on the lateral aspect of the knee with the axis of rotation at the lateral femur condyle. Because VAG signal might be distorted by the electrogoniometer placing, which could generate noise signal, this procedure was only used during determining of experimental condition before relevant tests.

The vibroarthrographic signals generated by motion of knee joint were collected by a piezoelectric accelerometer (type 4513B-002, Brüel & Kjær Sound & Vibration Measurement A/S, Denmark). The signal received by a transducer was passed on the input of a low-noise measuring amplified series Nexus by Brüel & Kjær company. A computer equipped with a measuring card type CH 3160 (Acquitek, France) and specialized AcquiFlex software were used for registration of the signals. Data were recorded in the periodicity between 0.7 and 1000 Hz at sampling frequency 10 kHz and then filtered using a fourth-order zero-phase Butterworth band-pass digital filter with cutoff frequencies at 50 Hz and 1000 Hz.

### 2.3. Signal Parameters

The variability of the VAG signal was assessed by computing the mean-squared values of an obtained signal in fixed-duration segments of 5 ms each and then computing the variance of the values of the parameter over the entire duration of the signal (VMS) [14].

The frequency characteristics of the VAG signal were examined by a short-time Fourier transform analysis [15]. The short-time spectra were obtained by computing the discrete Fourier transform of segments, 150 samples each, Hanning window, and 100 samples overlap of each segment. The spectral activity was analyzed by summing spectral power of the VAG signal in two bands: 50–250 Hz (P1) and 250–450 Hz (P2) [16].

In order to characterize range of significant amplitude values, an entropy (*H*) measure was used [10]. The measure is based upon the probability density function (PDF) of the given signal, denoted by *px*(*xl*), with *xl*, *l* = 0,1, 2,…, *L* − 1, representing the *L* = 248 bins used to represent the range of the values of the signal *x*. Entropy is a measure of the nature and spread of the PDF and is defined as a sum of *px*(*xl*) multiplied by log⁡⁡*px*(*xl*).

### 2.4. Statistical Analysis

Normality of the distribution was assessed with the Shapiro-Wilk test. Because of skewed distribution of VMS, P1, and P2 parameter values, they were analyzed after logarithmic transformation.

Evaluation of all dependent variables was performed with analysis of variance (ANOVA), and then for post hoc comparisons between mean values the Tukey test was used. For correlation between age and VAG parameter values Pearson* r* tests were performed. The level of significance was set at *P* < 0.05. Statistical analyses were performed using Statistica v.10 (StatSoft, Inc., OK, USA).

## 3. Results

**Figure 1:**
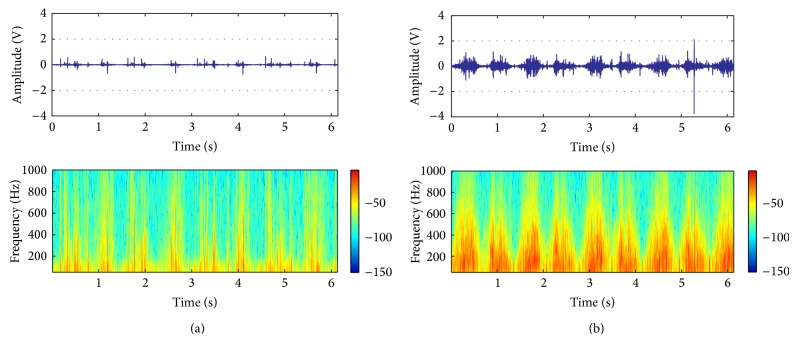
Course of the VAG signal and time-frequency analysis representatives for persons in 3th decade of life (a); 7th decade of life (b).

**Figure 2:**
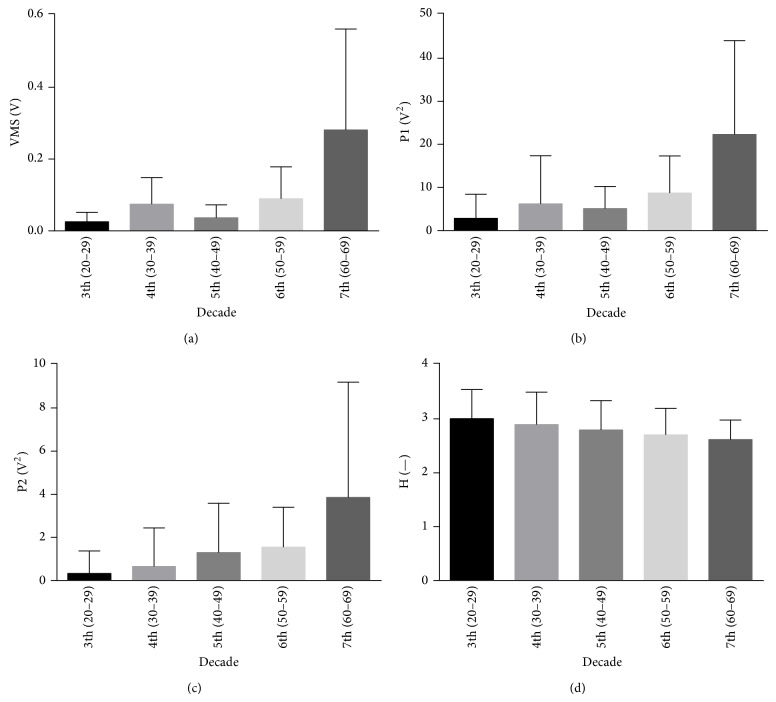
Mean and standard deviations of VAG signal parameters in the further decades of life.

In Figure 1 representative vibroarthrographic signal courses registered for individuals in 3th decade (between the ages of 20 and 29) and 7th decade (60–69 years) of life were shown, and the values of signal parameters achieved for all tested groups were presented in Figure 2. The parameters VMS, P1, and P2 possess the lowest values in vicenarians and the highest in the oldest individuals' group. Additionally, in the subsequence decades of life (from 4th to 7th) statistically significant higher levels of the discussed parameters in comparison to the youngest group were found (Table 2). The statistical analysis did not indicate any differences in patients who are between the ages of 30 and 59 when the parameters VMS and P1 were considered. In those cases in 7th decade of life, moreover, an about 3-fold higher value of the parameters VMS and P1 in comparison to those in 6th decade was observed. On the other hand, for P2 and additionally for *H* parameter the linear trends (increase and decrease, resp.) were achieved (Figure 2). But, in the decreasing of *H* level only one statistically significant difference (between vicenarians and sexagenarians) was observed (Table 2).

**Figure 3:**
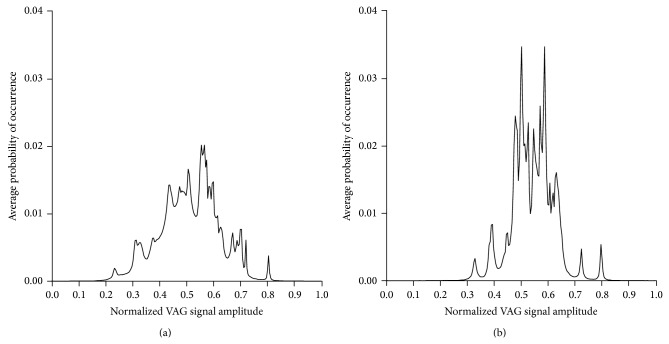
Histograms of VAG signals: derived from 3th decade subject (a) and derived from 7th decade subject (b). The amplitude has been normalized to the range [0,1].

Distributions of the normalized VAG signal amplitude were shown in Figure 3, where an average probability of occurrence as a higher level for cases in 7th decade of life than in 3th decade of life was presented, especially in 0.4–0.65 range of amplitude.

Because of continuous feature of the senescence process, the correlation of particular age of examined individuals and VAG signal parameters values was investigated. This analysis confirms that the parameters VMS, P1, and P2 positively correlate with age, and for left and right knees correlation coefficients are similar. For entropy, negative correlation was observed, which is in accordance with the age-related decreasing trends of *H* parameter in both analyzed knees (Table 3).

## 4. Discussion

VAG method relies on an acceleration sensor used for collecting of the mechanical vibrations generated by the articular surfaces movement [17]. An interpretation of the vibroarthrographic signals induced during joint motion was in the area of investigators interest since the early XX century. Then, Blodgett [18] using stethoscopic examination showed more grating and cracking of the knee joint sounds being emitted by older subjects in comparison to younger. Recently, Shark et al. [19] described a trend of increase signal magnitude in strong correlation with both knee age and condition including osteoarthritis occurrence. This phenomenon is associated, probably, with the age-related deterioration of cartilage mechanical properties, and as a consequence provides limited ability to the friction reduction. Moreover, it has been shown that the articular surfaces covered by defected cartilage require higher energy for joint motion [20]. This* In vitro* sheep model also documents that a decline of the friction properties of articular surfaces was correlated with cartilage defect size. The defects of cartilage appear to age as a consequence of the mechanical wear and tear and physiological aging process [4]. The age-dependent changes in chondrocytes biology based on the reduced number and impairment of functional ability, what is associated with cellular senescence including reduced synthesis activity and weakened response to the growth factors. Due to these, an accumulation of either degraded or aggregated matrix specific proteins (e.g., collagens and proteoglycans) and reduction of water content are observed [21]. Additionally, in rabbit model it has been shown that the limited viscoelastic properties of the chondrocytes and pericellular matrix affect the biomechanical environment of the knee joint cartilage degeneration that occurs in physiological senescence [22].

The above discussed mechanisms contribute to cartilage disorders and may lead to an alteration of VAG signals pattern. To date, the majority of published papers are focused on the normal and abnormal joints and confirm the phenomenon that degenerative articular surfaces create vibroarthrographic signals characterized by higher variability in comparison to healthy cartilage [23, 24].

However, our results were obtained for healthy asymptomatic knees with undetectable either structural or functional disturbances. This selection of participants provides us with analysis of physiological (nonpathological) process of cartilage senescence, and additionally a sufficient abnormal knee exclusion protects us against disorder-related “artifacts” in the VAG signals course.

Our results indicate age-related VAG signals hallmarks in the rising of the parameters values according to further decades of life. It seems that increase of VMS and frequency parameters (P1 and P2) could be associated with degenerative changes in cartilage, primary symptoms of the locomotor system aging. This finding is expected due to postulated hypothesis that increase of VAG signal values corresponds to decline in both the cartilage condition and quality of joint motion [19, 25].

Interestingly, a lack of the changes in the variability and frequency in range 50–250 Hz of recorded VAG signals between three decades of life (from 4th to 6th) seems to be associated with similar style of both private and professional lives of individuals who are between the ages of 30 and 59 [26]. This phenomenon leads to the virtually similar condition of the articular cartilage as well as PFJ quality motion. Nevertheless, the physiological senescence-related changes in cartilaginous tissue morphology successively impair functional integrity in middle adolescence. It is particularly presented as a greater participation of vibrations above 250 Hz of frequency.

The significant increases of the VMS and frequency parameters (P1 and P2) values between quinquagenarian and sexagenarians could be explained as a life style change associated with transitioning to the retirement. It seems that the decline of physical and social activity contributes to rapid acceleration of the aging process [26].

Surprisingly, an entropy parameter possesses the age-related decreasing trend, where the highest level in the youngest individuals' group but the lowest in the oldest was observed. However, Rangayyan and Wu [10] described the higher entropy values in abnormal in comparison to normal knee joint group. Entropy is defined as one of the measures describing variability and characterizing level of the disorder VAG course. Young age-related articular surfaces are covered by viscoelastic cartilage which contributes to generating low amplitude of the signals, but it is likely that chaotic peaks will occur and could lead to higher entropy values in VAG signals analysis. However, more frequent vibrations generated by articular surfaces movement are associated with senescence-related changes within the hyaline cartilage. The reason of this is that the higher friction properties provide more systematic course of the VAG signals and decreased values of entropy. In our opinion, the discrepancy between the results of Rangayyan and Wu [10] and results presented herein could be explained as a different background of cartilage function impairment in pathological and physiological (senescence-depended) processes which lead to varying entropy parameter values. However, it seems to be difficult to find a clear borderline between pathological and senescence-dependent factors affecting VAG parameters conducting, especially when our findings in this study should be considered exploratory.

## 5. Conclusions

A quality of joint motion is associated with articular cartilage condition, which is altering during senescence. The results presented herein are based on the VAG signals analysis of PFJ motion. We show that values of the obtained parameters correspond to the age of the participants. It could be discussed as an age-related change in hyaline cartilage structure, which influences the VAG signals pattern. In the best of our knowledge, this is the first report indicating age-related changes in the vibroarthrographic signal course in particular decades of human life. It seems that the VAG could be helpful in advanced assessment (objective, noninvasive, and low cost) of articular surfaces condition and quality of joint motion.

## Figures and Tables

**Table 1:** Anthropometric characteristics of the participants.

Parameters	Decades of life (years old)				
	3th (20–29)	4th (30–39)	5th (40–49)	6th (50–59)	7th (60–69)
Number of subjects (M/F)	60 (26/34)	56 (24/32)	40 (17/23)	31 (13/18)	33 (13/20)
Age (yrs)	23.8 ± 2.4	34.9 ± 3.0	45.5 ± 2.7	55.0 ± 3.0	64.8 ± 2.9
Mass (kg)	69.3 ± 11.8	71.5 ± 14.0	73.5 ± 12.1	74.6 ± 11.5	72.9 ± 9.0
Height (cm)	170.4 ± 7.5	169.3 ± 8.5	168.5 ± 8.9	167.0 ± 6.7	164.2 ± 8.2
BMI	23.8 ± 3.1	24.9 ± 4.5	25.9 ± 3.7	26.7 ± 3.4	27.1 ± 3.4

Values are expressed by mean ± SD.

**Table 2:** Statistical differences (*P* values) between particular decades of life in VAG signal parameters.

Compared groups	VMS	P1	P2	*H*
3th vs 4th	0.001	0.000	0.005	0.807
3th vs 5th	0.000	0.000	0.000	0.397
3th vs 6th	0.000	0.000	0.000	0.176
3th vs 7th	0.000	0.000	0.000	0.023
4th vs 5th	0.583	0.576	0.002	0.921
4th vs 6th	0.113	0.093	0.000	0.634
4th vs 7th	0.000	0.000	0.000	0.201
5th vs 6th	0.799	0.760	0.865	0.965
5th vs 7th	0.001	0.000	0.001	0.634
6th vs 7th	0.054	0.010	0.026	0.956

VMS, variance of the mean squares; P1 and P2, power spectral density bands: 50–250 Hz and 250–450 Hz, respectively; *H*, entropy.

**Table 3:** Correlations between age of particular participants and VAG parameters.

	VMS^*^	P1^*^	P2^*^	*H* ^*^
Left knee	0.53	0.58	0.60	−0.18
Right knee	0.52	0.54	0.64	−0.28

^*^Results are expressed as correlation coefficients; all factors were statistically significant (*P* < 0.05).

VMS, variance of the mean squares; P1 and P2, power spectral density bands: 50–250 Hz and 250–450 Hz, respectively; *H*, entropy.

## References

[1] Christensen K., Doblhammer G., Rau R., Vaupel J. W. (2009). Ageing populations: the challenges ahead. *The Lancet*.

[2] Shively C. A., Willard S. L., Register T. C. (2012). Aging and physical mobility in group-housed Old World monkeys. *Age*.

[3] Mau-Moeller A., Behrens M., Lindner T., Bader R., Bruhn S. (2013). Age-related changes in neuromuscular function of the quadriceps muscle in physically active adults. *Journal of Electromyography and Kinesiology*.

[4] Li Y. P., Wei X. C., Zhou J. M., Wei L. (2013). The age-related changes in cartilage and osteoarthritis. *BioMed Research International*.

[5] Berenbaum F. (2013). Osteoarthritis as an inflammatory disease (osteoarthritis is not osteoarthrosis!). *Osteoarthritis and Cartilage*.

[6] Felson D. T. (2013). Osteoarthritis as a disease of mechanics. *Osteoarthritis and Cartilage*.

[7] Song C.-Y., Lin J.-J., Jan M.-H., Lin Y.-F. (2011). The role of patellar alignment and tracking in vivo: the potential mechanism of patellofemoral pain syndrome. *Physical Therapy in Sport*.

[8] Kijowski R., Blankenbaker D., Stanton P., Fine J., Smet A. (2006). Correlation between radiographic findings of osteoarthritis and arthroscopic findings of articular cartilage degeneration within the patellofemoral joint. *Skeletal Radiology*.

[9] Gudbergsen H., Lohmander L. S., Jones G. (2013). Correlations between radiographic assessments and MRI features of knee osteoarthritis—a cross-sectional study. *Osteoarthritis and Cartilage*.

[10] Rangayyan R. M., Wu Y. F. (2008). Screening of knee-joint vibroarthrographic signals using statistical parameters and radial basis functions. *Medical and Biological Engineering and Computing*.

[11] Kim K. S., Seo J. H., Kang J. U., Song C. G. (2009). An enhanced algorithm for knee joint sound classification using feature extraction based on time-frequency analysis. *Computer Methods and Programs in Biomedicine*.

[12] Mascaro B., Prior J., Shark L.-K., Selfe J., Cole P., Goodacre J. (2009). Exploratory study of a non-invasive method based on acoustic emission for assessing the dynamic integrity of knee joints. *Medical Engineering and Physics*.

[13] Crema M. D., Guermazi A., Sayre E. C. (2011). The association of magnetic resonance imaging (MRI)-detected structural pathology of the knee with crepitus in a population-based cohort with knee pain: the MoDEKO study. *Osteoarthritis and Cartilage*.

[14] Rangayyan R. M., Wu Y. (2009). Analysis of vibroarthrographic signals with features related to signal variability and radial-basis functions. *Annals of Biomedical Engineering*.

[15] Moussavi Z. M. K., Rangayyan R. M., Bell G. D., Frank C. B., Ladly K. O., Zhang Y.-T. (1996). Screening of vibroarthrographic signals via adaptive segmentation and linear prediation modeling. *IEEE Transactions on Biomedical Engineering*.

[16] Baczkowicz D., Krecisz K. (2013). Vibroarthrography in the evaluation of musculoskeletal system—a pilot study. *Ortopedia Traumatologia Rehabilitacja*.

[17] Mu T., Nandi A. K., Rangayyan R. M. (2008). Screening of knee-joint vibroarthrographic signals using the strict 2-surface proximal classifier and genetic algorithm. *Computers in Biology and Medicine*.

[18] Blodgett W. E. (1902). Auscultation of the knee joint. *Boston Medical and Surgical Journal*.

[19] Shark L.-K., Chen H., Goodacre J. (2011). Knee acoustic emission: a potential biomarker for quantitative assessment of joint ageing and degeneration. *Medical Engineering & Physics*.

[20] Lorenz A., Rothstock S., Bobrowitsch E. (2013). Cartilage surface characterization by frictional dissipated energy during axially loaded knee flexion—an in vitro sheep model. *Journal of Biomechanics*.

[21] Martin J. A., Buckwalter J. A. (2001). Roles of articular cartilage aging and chondrocyte senescence in the pathogenesis of osteoarthritis. *The Iowa Orthopaedic Journal*.

[22] Duan W.-P., Sun Z.-W., Li Q. (2012). Normal age-related viscoelastic properties of chondrons and chondrocytes isolated from rabbit knee. *Chinese Medical Journal*.

[23] Rangayyan R. M., Oloumi F., Wu Y., Cai S. (2013). Fractal analysis of knee-joint vibroarthrographic signals via power spectral analysis. *Biomedical Signal Processing and Control*.

[24] Tanaka N., Hoshiyama M. (2012). Vibroarthrography in patients with knee arthropathy. *Journal of Back and Musculoskeletal Rehabilitation*.

[25] Leszko F., Zingde S., Argenson J. N. (2013). Vibroarthrography as a potential non-invasive diagnostic tool: application to articular cartilage condition assessment. *Journal of Bone and Joint Surgery*.

[26] Calvo E., Sarkisian N., Tamborini C. R. (2013). Causal effects of retirement timing on subjective physical and emotional health. *Journals of Gerontology—Series B Psychological Sciences and Social Sciences*.

